# Investigation of oxidative stress and dietary habits in Mongolian people, compared to Japanese people

**DOI:** 10.1186/1743-7075-3-21

**Published:** 2006-06-07

**Authors:** Fumio Komatsu, Yasuo Kagawa, Mitsuru Sakuma, Terue Kawabata, Yoshinori Kaneko, Dugee Otgontuya, Ulziiburen Chimedregzen, Luvsanbazar Narantuya, Baatar Purvee

**Affiliations:** 1 High Technology Research Center, Kagawa Nutrition University, 3-9-21 Chiyoda, Sakado, Saitama, 350-0288, Japan; 2 Public Health Institute, Ministry of Health of Mongolia, Peace Avenue-17, Ulaanbaatar-49, Mongolia; 3 Khuvsgul General Hospital, Khuvsgul Aimag, Mongolia

## Abstract

**Background:**

The average life span of Mongolians is 62 years for males and 69 years for females. This life span is about 16 years shorter than that of Japanese. Mongolian people generally eat meat, fat and diary products but less vegetables or fruit. Thus, we investigated the state of oxidative stress and dietary habits of Mongolians.

**Methods:**

The investigation was performed in Murun city in the northwest area of Mongolia. A total of 164 healthy subjects (24–66 y) were enrolled. As a marker of reactive oxygen species, the levels of reactive oxygen metabolites (ROM) were measured using the d-ROM test. Interviews about dietary habits were performed using the Food Frequency Questionnaire established by the Kagawa Nutrition University.

**Results:**

ROM levels were 429.7 ± 95.2 Carr U for Murun subjects, whereas Japanese people (n = 220, 21–98 y) showed 335.3 ± 59.8 (p < 0.001). The levels of serum malondialdehyde-modified low-density lipoprotein-cholesterol and urinary 8-hydroxydeoxyguanosine were also high. ROM levels correlated with body fat ratio and inversely correlated with handgrip strength. Handgrip strength in the subjects over 45 years decreased more rapidly than that of age-matched Japanese. Murun subjects ate larger amounts of meat, fat, milk and flour and dairy products than Japanese, but less vegetables or fruit. Serum vitamin A and E levels were the same as Japanese references, but vitamin C levels were lower.

**Conclusion:**

Murun subjects may be in high oxidative stress, which may have a relationship with early ageing and several diseases, ultimately resulting in their short life span. In order to increase antioxidant capacity and suppress overproduction of ROM, antioxidant food intake is recommended.

## Background

Mongolia has a population of 2,504,000. The average life span is 62 years for males and 69 years for females (WHO World Health Report, 2005). This life span is shorter than that of Japanese (male, 79 y; female, 85 y) [1]. In 2002, we investigated the health status of the residents in Ulaanbaatar, the capital of Mongolia, and found that not only communicable diseases, such as viral hepatitis, but also non-communicable diseases were prevalent in this country [2]. Reasons for this may be due to several factors, including difficult living conditions, physical labor, economic hardship, smoking, alcohol drinking and dietary habits. Diets mainly consist of meat, flour and dairy products. The subjects in Ulaanbaatar seldom ate vegetables or fruit, although urbanized subjects took high amount of fruit. Vegetables and fruits are advantageous because of their ability to suppress oxidative stress [3,4].

Oxidative stress is induced by the overproduction of reactive oxygen species (ROS), which oxidize lipids, proteins and DNA, and lead to cell membrane destruction, resulting in several diseases. Atherosclerosis, heart disease, stroke, diabetic vascular disease, cancer and even early aging are thought to be induced by oxidative stress [5,6]. On the other hand, the human body generally has antioxidant capacity (AOC). AOC plays an important role in the suppression of ROS overproduction and protects cells from oxidative stress. AOC is comprised of endogenous compounds (bilirubin, uric acid, superoxide dismutases, catalase, glutathione peroxidase etc.) and exogenous compounds (carotenoids, tocopherols, ascorbate, bioflavonoids etc) [7]. The exogenous compounds are provided by foods, in particular, vegetables and fruits [3,4]. These findings led us to investigate the state of oxidative stress in Mongolians.

Direct measurement of ROS and free radicals in a standard laboratory is difficult owing to their biochemical instability. Recently, a method of measuring reactive oxygen metabolites (ROM) in blood has been developed. This method is the d-ROM test, which uses the Free Radical Analytical System (FRAS, Diacron, Grosseto, Italy) [8,9]. The main component of ROM is hydroperoxides. Despite fair oxidant power, hydroperoxides in the blood are relatively stable compared to its parent free radicals, and therefore, the level can be detected. Hydroperoxides cause cell death and tissue damage. Carratelli et al. [8] noted that hydroperoxides are associated with a cellar dysfunction in Down syndrome. Yagi et al. [10] stated that administration of hydroperoxides into a rabbit induced a marked lesion in the intima of the aorta, which is an initial event in atheroscrelosis. This test has already been recognized as useful for the evaluation of oxidative stress in the body [11,12]. Because the Analyzer (FRAS) is compact, we could carry it to Mongolia.

In July of 2005, we investigated the state of oxidative stress by measuring ROM levels in the people of Murun city, a northwest area of Mongolia, because of information that the people seldom eat vegetables or fruit. In this report, we describe the dietary habits of people in Murun city and their blood levels of ROM, and compare them with levels in Japanese people.

## Methods

### Subjects

In July 2005, we investigated Mongolians in Murun city, which is a central town of Khuvsgul Prefecture, located in northwest Mongolia, 700 km from Ulaanbaatar. As shown in Table 1, a total of 164 healthy subjects, ranging in age from 24 to 66 years (72 males and 92 females), were randomly assembled by native researchers. The investigation was performed at the Khuvsgul General Hospital. The protocol of this study was approved by the Ethics Committee of the Kagawa Nutrition University and the Public Health Institute, Ministry of Health of Mongolia, according to the principles of the Declaration of Helsinki (Edinburgh, October 2000). The subjects gave written informed consent before the study. Because we also investigated the health status of Ulaanbaatar people in September 2002 [2], we describe some of the results in this report. The Ulaanbaatar subjects (n = 256) were assembled from two areas of town and were designated Group A and Group B, as shown in later. Furthermore, we compare ROM levels of Murun subjects with those of healthy Japanese subjects (n = 220). Japanese subjects were sampled in the Tokyo area. They included elderly subjects aged 70 to 98 years living in nursing facilities. Habitual heavy smokers, heavy alcohol users, aerobics trainers and contraceptive pill users were not included in the Japanese sample because they generally show high ROM levels.

**Table 1:** The number of Murun, Ulaanbaatar (Group A and B) and Japanese Subjects

	Murun Subjects (24–66 y)		Group B (25–75 y)		Ulaanbaatar Subjects Group A (25–75 y)		Japanese Subjects (20–98 y)	
	n	Age (y)	n	Age (y)	n	Age (y)	n	Age (y)
Total	164	44.7 ± 10.4	142	48.4 ± 10.8	114	48.2 ± 10.2	220	49.4 ± 22.8
								
Males	72	46.1 ± 10.1	71	49.8 ± 14.2	57	49.7 ± 14.3	96	48.4 ± 15.3
Females	92	44.1 ± 10.5	71	47.2 ± 13.1	57	47.2 ± 13.1	124	48.4 ± 26.9

Investigation of Ulaanbaatar subjects was performed in 2002.

### Investigation of daily food intake

Daily food intake was investigated using the Food Frequency Questionnaire developed at the Kagawa Nutrition University. Subject interviews were performed by well-trained native and Japanese food specialists, taking 15 to 20 minutes for each subject. In order to confirm the quantity of foods, pictures and photos of pan, pot, ladle, dish and cooked foods were shown to the subjects. The food specialists calculated the total food intake using the Standard Tables of Food Composition [13].

### Anthropometric measurements

Anthropometric measurements and physical examinations, including blood pressure (BP), height, body weight (BW), body mass index (BMI), body fat ratio (%BF) and handgrip strength, were performed by medical doctors and licensed medical technicians. BMI was calculated from the formula, weight (kg)/height (meters)^2^. Percent BF was measured using a bioelectrical impedance meter (Professional Body Composition Analyzer TBF 110, Tanita Co., Tokyo, Japan). Handgrip strength was measured using the Dynamo Meter T-1780 (TOEI LIGHT, Tokyo, Japan).

### Measurement of Reactive Oxygen Metabolites (ROM)

To measure ROM, the d-ROM test was performed using the FRAS4, according to the analysis procedures. In brief, a 20 *μ*L blood sample (from a fingertip) and 1 mL of buffered solution (R2 reagent of kit, pH 4.8) were gently mixed in a cuvette, and then 10 *μ*L of chromogenic substrate (R1 reagent of kit) was added into the cuvette. After mixing, the cuvette was centrifuged for 60 seconds at 37°C and immediately incubated in the thermostatic block of this analyzer for 5 min at 37°C. Then, the 505 nm absorbance was recorded. The measurement unit was expressed as Carr U. It has been established that 1 Carr U corresponds to 0.08 mg/dL H_2_O_2_. Reference values indicated by the manufacturer (Diacron) are from 250 to 300 Carr U; values higher than 300 Carr U suggest oxidative stress [8,9].

### Measurements of serum biochemical markers

To measure serum biochemical markers, peripheral blood was drawn following overnight fasting, and the serum was obtained by centrifugation (1500 g for 15 min at 4°C), put into plastic tubes, rapidly frozen, transferred to Tokyo and stored under -80°C until the analyses were done. Albumin, total cholesterol (T-chol), low density lipoprotein cholesterol (LDL), high density lipoprotein cholesterol (HDL), triglyceride (TG), alanine aminotransferase (ALT), blood urea nitrogen (BUN), iron and uric acid were measured by well-established routine procedures. Folic acid was measured by a chemiluminescence enzyme immunoassay, and vitamin A (tocopherol) and vitamin C (ascorbate) by a high-performance liquid chromatography method. For the measurement of vitamin C, an antioxidant agent was added to the samples just after blood drawing. Vitamin E was measured by a fluorimetric method, malondialdehyde-modified LDL (MDA) by an enzyme-linked immunosorbent assay (ELISA) [14] and urinary 8-hydroxy-2'-deoxyguanosine (8-OHdG) by using an ELISA kit (Japan Institute for Control of Aging, Shizuoka, Japan) [15]. As a marker of endogenous oxidative defense system, superoxide dismutase (SOD) activity was measured by the nitrite method [16]. The presence of hepatitis B virus (HBV) was evaluated by detection of HBsAg using a commercial assay kit (Mycell HBsAg, Institute of Immunology Co. Ltd, Tokyo, Japan) and the presence of hepatitis C virus (HCV) using a commercial assay kit (Anti-HCV, Abbott, Dainabot, Tokyo, Japan). These measurements were performed in a commercial laboratory (the SRL Inc., Tokyo, Japan).

The results were expressed as mean ± standard deviation (M ± SD). For evaluation of the difference, *t*-test was used, and a p value < 0.05 was considered to be statistically significant.

## Results

### 1. Environment and socioeconomic status

Murun city is in a rural area. The temperature in summer rises to 30°C, but it goes below -40°C in winter. The land is flat, but there is no farming. The population is 30,000. A total of 164 subjects were enrolled (Table 1). All of them appeared to be healthy and sufficiently nourished. They lived with five to seven family members in houses or narrow gers (tents), which were made in their individual enclosures. Mean average monthly income of each family was 121,412.5 ± 100,527.3 tugrug (1200 tugrug/US dollar, July 2005). However, two thirds of the families earned less than 90,000 tugrug. After a free economy system was put in place, the gap between rich and poor widened. Recently new workers who had just graduated in Ulaanbaatar usually earn 70,000 tugrug.

In 2002, when we investigated the health status of the people of Ulaanbaatar, Group A subjects (n = 142) earned 158,605.6 ± 152,770.9 tugrug and Group B subjects (n = 114) earned 67,828.3 ± 30,784.5 tugrug (p < 0.01). Group A people lived in comfortable houses or urbanized apartments in the central area of Ulaanbaatar, and their families were comprised of three to five members. They drank public service water and used flush toilets. Meanwhile, Group B people (who had moved to Ulaanbaatar from nomadic areas 10 to 20 years ago) lived in narrow gers on the outskirts of Ulaanbaatar, with seven to nine people per family. They drank well water (there was one well in the center of each ger settlement), and used a drop hole toilet. Murun residents also drank well water and used a drop hole toilet. The living environment and economic conditions of Murun residents were similar to those of Ulaanbaatar Group B subjects.

### 2. Daily food intake

**Figure 1:**
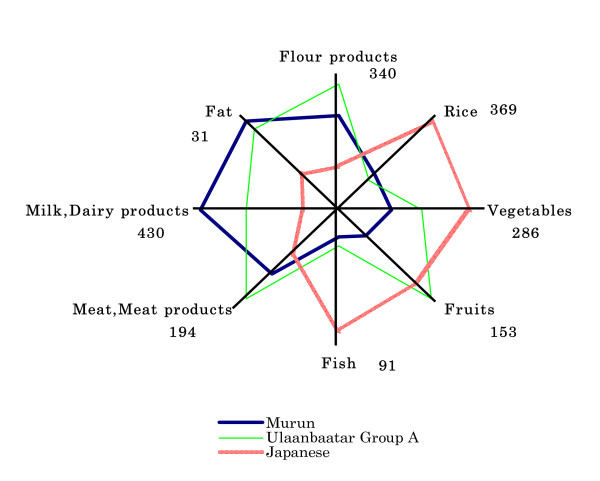
Comparison of daily food intake (g/day) among Murun, Ulaanbaatar Group A and Japanese subjects. Murun subjects seldom eat vegetables or fruit. Ulaanbaatar Group A subjects lived by urbane-styles and ate high amounts of fruits.

Daily food intake of Murun subjects (in 2005), Ulaanbaatar Group A and Group B people (in 2002) and Japanese people (in 2003) are shown in Table 2. Mongolian subjects ate rice, wheat flour products, potatoes, vegetables, meat and meat products, milk, dairy products, fruits, fat and tea. Main characteristics were as follows: Group A subjects ate a Western-style diet, including adequate amounts of vegetables and fruits. Group B subjects ate a traditional diet. They ate only small amounts of vegetables and fruits, because vegetables and fruits were imported from China and Korea and were expensive for them. Murun subjects had a diet similar to Group B subjects, but they seldom ate vegetables or fruit. In summer, Murun subjects ate only a small amount of cabbage, carrots and onions in addition to potatoes. They seemed not to have a habit of vegetable intake. However, they ate large amounts of snacks, sweetened buns and pastries. Some of these foods were imported from Russia. In summer, Mongolian people generally drink koumiss, but koumiss was rare in Murun city because it was not produced in this area. They did not take any supplements. They used large amounts of salt for cooking. They routinely drank vodka, which has high alcohol content. Their dietary habits were very different from those of Ulaanbaatar Group A and Japanese people, as shown in Fig. 1.

**Table 2:** Comparison of Daily Food Intake among Ulaanbaatar (Group A and B), Murun and Japanese Subjects.

							(g/day)
	**Ulaanbaatar**			**Murun**			**Japanese**

	Group A n = 142	Group B n = 114	1)	n = 164	2)	3)	n = 7,164

**Rice**	**67.2 ± 50.4**	**50.5 ± 33.1**	**	**68.1 ± 55.5**		***	**369.4 ± 197.9**
Rice	57.1 ± 42.6	47.7 ± 30.9	*	66.1 ± 54.9		***	
Millet	3.4 ± 18.0	1.4 ± 4.8		0.0 ± 0.4	*	***	
Buckwheat	1.4 ± 3.6	0.6 ± 3.6		0.5 ± 2.2	*		
Farina	5.3 ± 16.7	0.9 ± 4.2	**	1.5 ± 6.3	*		
**Flour products**	**338.1 ± 140.0**	**308.9 ± 142.0**		**264.1 ± 117.5**	***	**	**102.9 ± 114.1**
White bread	102.7 ± 63.0	85.9 ± 61.0	*	69.3 ± 40.6	***	*	
Black bread	12.2 ± 23.7	2.5 ± 6.7	***	4.9 ± 11.8	***	*	
Barley flour	2.5 ± 5.6	4.1 ± 8.6		2.3 ± 6.2			
Black flour	5.7 ± 24.7	14.8 ± 35.1	*	7.8 ± 27.7			
Other flour	113.2 ± 72.5	100.9 ± 60.0		119.3 ± 86.3		*	
Noodle	20.2 ± 27.3	8.9 ± 10.9	***	19.6 ± 25.7		***	
Biscuits	23.4 ± 36.9	23.6 ± 34.4		27.8 ± 44.0			
Fried cake	37.7 ± 56.5	62.7 ± 77.3	**	13.0 ± 31.3	***	***	
Others	19.5 ± 42.2	5.9 ± 16.5	**	1.6 ± 7.8	***	*	
**Potatoes**	**94.9 ± 57.0**	**70.1 ± 50.0**	***	**59.1 ± 43.1**	***		**57.6 ± 70.7**
**Vegetables**	**226.2 ± 184.3**	**108.1 ± 83.3**	***	**72.2 ± 44.0**	***	***	**286.4 ± 170.9**
Carrot	27.4 ± 21.8	15.7 ± 15.0	***	14.3 ± 14.0	***		
Turnip	19.1 ± 22.8	13.2 ± 14.1	*	2.5 ± 7.5	***	***	
Green onion	13.8 ± 13.4	11.2 ± 6.9		22.7 ± 14.9	***	***	
Garlic	1.0 ± 1.7	0.4 ± 1.1	***	0.3 ± 0.7	***		
Cucumber	27.7 ± 39.2	6.0 ± 12.4	***	4.9 ± 10.5	***		
Tomato	30.7 ± 45.4	8.5 ± 20.5	***	2.8 ± 8.2	***	***	
Sugar beet turnip	4.6 ± 15.0	1.1 ± 3.6	*	0.0 ± 0.0	***	***	
Spinach	0.2 ± 0.8	0.1 ± 0.8		0.0 ± 0.1	*		
Lettuce	1.8 ± 8.0	0.3 ± 1.6	*	0.0 ± 0.2	**		
Pepper	11.8 ± 25.6	2.0 ± 6.7	***	0.4 ± 1.5	***	*	
Kelp	0.7 ± 2.4	1.0 ± 9.4		0.1 ± 0.4	**		
Cabbages	57.5 ± 53.3	39.5 ± 38.5	**	16.9 ± 20.4	***	***	
Mixed salad	24.5 ± 32.3	9.1 ± 14.7	***	6.5 ± 9.8	***		
Green vegetables	1.3 ± 3.7	0.0 ± 0.1	***	0.8 ± 4.0		*	
Other vegetables	4.4 ± 18.0	0.1 ± 1.2	*	0.0 ± 0.0	***		
**Eggs**	**15.5 ± 21.0**	**4.4 ± 7.8**	***	**4.3 ± 8.4**	***		**37.3 ± 37.1**
**Meat & Meat products**	**193.9 ± 100.3**	**168.5 ± 79.1**	*	**122.7 ± 71.1**	***	***	**84.3 ± 73.1**
Mutton	87.0 ± 59.0	87.9 ± 48.6		68.0 ± 59.2	***	***	
Beef	3.1 ± 11.9	3.2 ± 8.7		22.5 ± 35.3	***	***	
Goat	56.4 ± 51.6	47.2 ± 41.8		9.2 ± 24.2	***	***	
Chicken	1.7 ± 4.9	0.6 ± 2.6	*	0.2 ± 1.1	***		
Pork meat	9.0 ± 21.4	1.4 ± 5.9	***	0.0 ± 0.4	***	*	
Stomach	5.5 ± 13.8	11.2 ± 25.3	*	5.3 ± 14.6		*	
horse	3.5 ± 11.4	5.7 ± 12.1		11.6 ± 28.3	***	*	
Camel	0.3 ± 2.1	0.3 ± 1.4				*	
Sausage, Ham	27.1 ± 35.9	10.8 ± 17.1	***	6.6 ± 14.9	***	*	
Others	0.9 ± 5.4	0.2 ± 1.4a		0.0 ± 0.2			
**Fish**	**9.4 ± 18.2**	**4.6 ± 11.4**	*	**5.5 ± 15.6**	*		**90.9 ± 81.3**
Fresh fish				4.6 ± 15.5	***	***	
Canned products	4.1 ± 9.7	1.7 ± 8.6		0.8 ± 2.3	***		
Others	5.4 ± 14.5	3.3 ± 8.4		4.6 ± 15.5	***	***	
**Milk & Dairy products**	**280.0 ± 237.1**	**244.0 ± 208.7**		**430.1 ± 293.4**	***	***	**95.7 ± 131.2**
Cow	102.7 ± 132.8	121.8 ± 116.8		198.3 ± 212.5	***	***	
Dry milk	0.3 ± 2.3	0.4 ± 1.0		0.0 ± 0.0		***	
Others milks	6.6 ± 26.2	2.6 ± 13.9		73.1 ± 152.5	***	***	
Cream thin layer	8.9 ± 22.2	7.9 ± 30.3		8.6 ± 13.9			
Liquid yogurt	76.5 ± 82.2	51.6 ± 60.6	**	119.1 ± 114.7	***	***	
Cheese/Mongol/	3.7 ± 9.4	2.5 ± 5.5		2.6 ± 5.8			
Others cheese	2.2 ± 5.6	0.2 ± 0.7	***	0.1 ± 0.3	***		
Dried milk curds	16.2 ± 34.6	10.2 ± 19.5		12.2 ± 17.3			
Koumiss	62.7 ± 131.0	47.7 ± 111.6		16.6 ± 43.1	***	***	
Others	0.2 ± 1.1	0.1 ± 0.5		2.2 ± 20.4			
**Fruits**	**152.5 ± 179.8**	**59.4 ± 86.1**	***	**29.5 ± 71.0**	***	***	**101.4 ± 127.3**
Apple	69.0 ± 81.1	34.5 ± 48.4	***	22.0 ± 55.0	***	*	
Orange & Lemon	7.3 ± 18.0	2.9 ± 14.1	*	0.7 ± 2.5	***		
Grapes	14.3 ± 33.5	6.0 ± 14.2	*	1.6 ± 6.4	***	***	
Banana	25.8 ± 50.1	6.5 ± 17.6	***	0.4 ± 3.0	***	***	
Other planted Fruits	20.7 ± 57.9	3.7 ± 10.3	**	2.4 ± 10.1	***		
Wild fruit's	15.9 ± 31.9	7.0 ± 15.6	**	2.4 ± 16.2	***	*	
**Fat**	**28.0 ± 28.8**	**33.7 ± 32.0**		**31.1 ± 21.5**			**11.2 ± 9.8**
Butter	10.0 ± 19.2	10.6 ± 21.9		12.4 ± 15.5			
Vegetqable oil	14.1 ± 15.5	13.7 ± 13.5		14.5 ± 12.0			
Animal oil	1.1 ± 3.3	5.0 ± 10.3	***	4.1 ± 10.3	***		
Melted butter	2.8 ± 7.4	4.3 ± 10.0		0.8 ± 3.1	***	***	
Others/marmot	0.0 ± 0.2	0.2 ± 1.9		0.0 ± 0.0			
**Sweets**	**17.1 ± 42.7**	**14.5 ± 26.7**		**14.7 ± 23.1**			**23.9 ± 47.6**
Chocolate	9.0 ± 28.3	5.6 ± 12.3		4.5 ± 8.1			
Others	8.0 ± 27.2	9.0 ± 18.0		10.5 ± 18.0			
**Seasoning**	**21.5 ± 17.5**	**18.9 ± 15.6**		**23.6 ± 16.9**		*	**103.7 ± 98.7**
Vinegar	2.2 ± 3.8	1.3 ± 2.7	*	0.2 ± 1.0	***	***	
Ketchup, Soy sauce	3.8 ± 5.6	2.2 ± 4.0	*	2.1 ± 3.8	***		
Sugar	11.6 ± 15.7	9.6 ± 10.9		13.2 ± 14.9		*	
Salt	3.2 ± 1.9	5.9 ± 9.9	**	8.4 ± 4.3	***	*	
Others	0.8 ± 1.7	0.1 ± 0.4	***	0.2 ± 0.9	***		
**Soft & Alcohol drinks**	**899.9 ± 406.0**	**827.0 ± 466.4**		**957.2 ± 519.2**		*	**684.3 ± 509.2**
Green tea	329.7 ± 241.7	459.0 ± 337.3	***	388.5 ± 233.7	*		
Black tea	133.4 ± 161.4	37.7 ± 85.7	***	82.8 ± 242.2	*	*	
Coffee	54.3 ± 82.7	23.0 ± 59.4	***	9.4 ± 25.6	**	***	
Soft drinks	87.0 ± 136.3	78.9 ± 127.4		102.0 ± 126.7			
Juice	50.5 ± 85.4	18.5 ± 34.8	***	8.8 ± 12.1	***	***	
Vodka	8.5 ± 17.4	9.7 ± 20.2		14.5 ± 19.4	*	**	
Beer and vine	31.7 ± 81.4	12.9 ± 30.4	*	5.4 ± 11.2	**	***	
Milk tea	203.7 ± 125.2	187.7 ± 245.2		329 ± 270.7	***	***	

Ulaanbaatar: Investigation was performed in 2002.

Japanese: in The National Health and Nutrition Survay in Japan, 2003, reported by Ministsry of Health, Labour and Welfare of Japan (ages of the subjects : 20–69 yrs)

1) : significance between Group A and Group B, 2) : between Group A and Murun, 3) : between Group B and Murun. * p < 0.05, ** p < 0.01, *** < p0.001.

### 3. Measurement of ROM levels

In order to evaluate oxidative stress, ROM levels were measured and compared to those of Japanese people (This test was not performed in Ulaanbaatar in 2002.)

**Figure 2:**
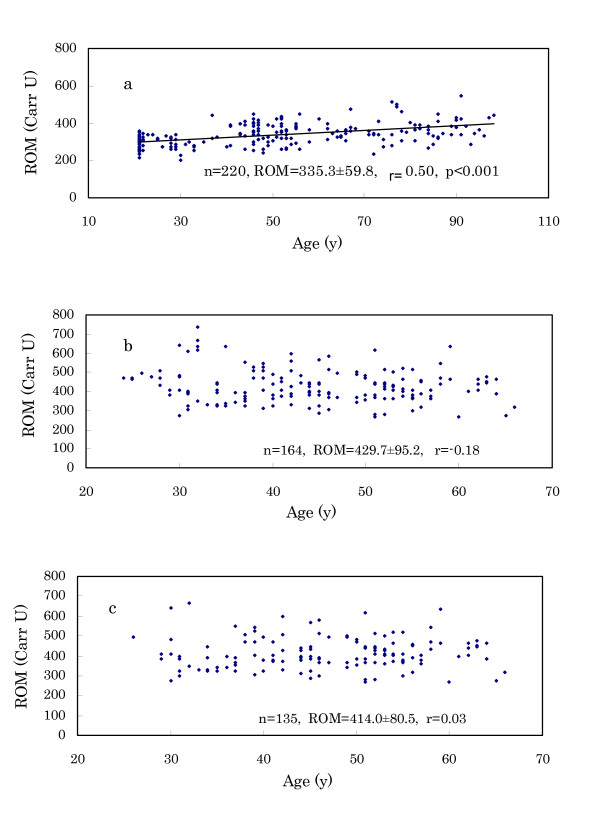
Relation of ROM levels with age. a: Japanese subjects, b: Murun subjects, c: Murun subjects without contraceptive pill users. Japanese young subjects showed low ROM levels, and the levels increased with age. But Murun subjects did not show this tendency. The levels were very high even in young ages.

(1) As shown in Fig. 2a and 2b, Japanese (n = 220) had ROM levels of 335.3 ± 59.8 Carr U, whereas Murun subjects' (n = 164) levels were 429.7 ± 95.2 Carr U. This difference is significant (p < 0.001). Young Japanese subjects exhibited low levels. In particular, female students aged 21 years (n = 41) demonstrated low levels (289.4 ± 33.3 Carr U). These levels were in desirable ranges indicated by the manufacturer (Diacron). The ROM levels of the Japanese increased significantly with age. But Murun subjects did not show such a tendency, because the levels were very high even in young subjects.

(2) Murun subjects were 24 to 66 years-old. Thus, ROM levels of age-matched Japanese were calculated. As shown in Table 3, Murun subjects still showed higher ROM levels compared to Japanese.

**Table 3:** Comparison of ROM Levels between Murun and Japanese Subjects.

Murun Subjects			Japanese Subjects		
(24–66 y)			(24–66 y)		
	n	ROM(Carr U)	n	Age(y)	ROM(Carr U)
Total	164	429.4 ± 95.2	115	45.9 ± 11.0	334.8 ± 53.7
Males	72	378.3 ± 65.7	80	44.9 ± 11.3	325.1 ± 5.14
Females	92	469.8 ± 95.8	35	48.1 ± 10.0	357.0 ± 52.8

Because Murun subjects were in 24–66 years, the results of Japanese subjects of 24–66 years were indicated. *** p < 0.001

(3) We have already noted that Japanese contraceptive pill users generally show high ROM levels, such as 500–600 Carr U. Thus, pill users were not included in the Japanese study. Habitual heavy smokers were also excluded from the Japanese subjects. In Murun subjects, ROM levels were compared between pill users and non-users. As shown in Table 4, the pill users showed significantly higher ROM levels than non-users. Among Murun subjects, not only pill users but also non-users showed higher ROM levels than age-matched Japanese females (p < 0.001). Therefore, it could be speculated that other factors in addition to pill use may increase the ROM levels in these subjects. In Fig. 2c, ROM levels in all except for pill users are shown. These findings suggest that Murun subjects may be in high oxidative stress compared to Japanese people.

**Table 4:** Comparisons of ROM Levels between Contraceptive Pill Users and Non-Users, Smokers and Non-Smokers, Alcohol Drinkers and Non-Drinkers, and Hepatitis Virus-Positive and Hepatitis Virus-Negative of Murun Subjects.

	n	Age (y)	ROM (Carr U)	
Females				
Pill users (24–44 yrs)	29	34.4 ± 6.3	502.7 ± 122.8	*
Non-users (26–66 yrs)	63	47.8 ± 9.3	454.7 ± 76.9	*
(Japanese Non-users)(26–66 yrs)	33	48.0 ± 9.8	356.2 ± 52.5	***
				
Males				
Smoker (31–64 yrs)	39	47.2 ± 9.0	381.8 ± 64.7	ns
Non-smoker (30–65 yrs)	33	44.9 ± 11.4	374.2 ± 67.5	ns
Females				
Smoker (59–66 yrs)	2	62.5 ± 4.9	475.0 ± 224.9	ns
Non-smoker (26–64 yrs)	61	47.3 ± 9.0	454.0 ± 72.5	ns
				
Males				
Drinkers (30–65 yrs)	61	46.2 ± 10.2	383.6 ± 67.0	ns
Non-drinkers (30–64 yrs)	11	46.2 ± 10.8	349.2 ± 50.5	ns
Females				
Drinkers (26–66 yrs)	53	47.5 ± 9.5	454.5 ± 79.2	ns
Non-drinkers (38–64 yrs)	10	49.5 ± 8.3	455.8 ± 66.8	ns
				
Males and Females				
HBsAg-Positive	22	46.3 ± 9.0	392.4 ± 68.0	ns
HBsAg-Negative	113	47.0 ± 10.0	418.2 ± 82.3	ns
				
Anti-HCV-Positive	41	49.1 ± 9.3	407.3 ± 75.9	ns
Anti-HCV-Negative	94	46.0 ± 9.9	416.9 ± 82.7	ns

ns : not significant, * p < 0.05, *** p < 0.001

In the following results, we excluded the pill users from the calculations because they may largely dominate the results.

(4) Concerning gender difference in ROM levels, females showed significantly higher levels than males (males (n = 72); 378.3 ± 65.7, females (pill non-users, n = 63); 454.7 ± 76.9 Carr U, p < 0.001).

(5) Because Japanese smokers and alcohol drinkers generally show higher ROM levels than controls, we compared ROM levels between smokers and non-smokers and between alcohol drinkers and non-drinkers in Murun subjects. As shown in Table 4, no significant differences were found between them. These findings suggest that factors other than smoking and drinking might have caused the elevation of ROM levels in Murun subjects.

(6) In Murun subjects, we found HBsAg in 16.3% and Anti-HCV in 30.4%. ROM levels were compared between HBsAg-positive and HBsAg-negative subjects and between anti-HCV-positive and anti-HCV-negative subjects. We did not find any differences between them (Table 4). This finding suggests that these viral infections did not affect ROM levels.

### 4. Anthropometric measurements

In Murun subjects, BP, height, BW, %BF and handgrip strength were measured and compared to age-matched Japanese standard references (reported by Japanese Ministry of Education, Culture, Sports, Science and Technology in 2003).

**Figure 3:**
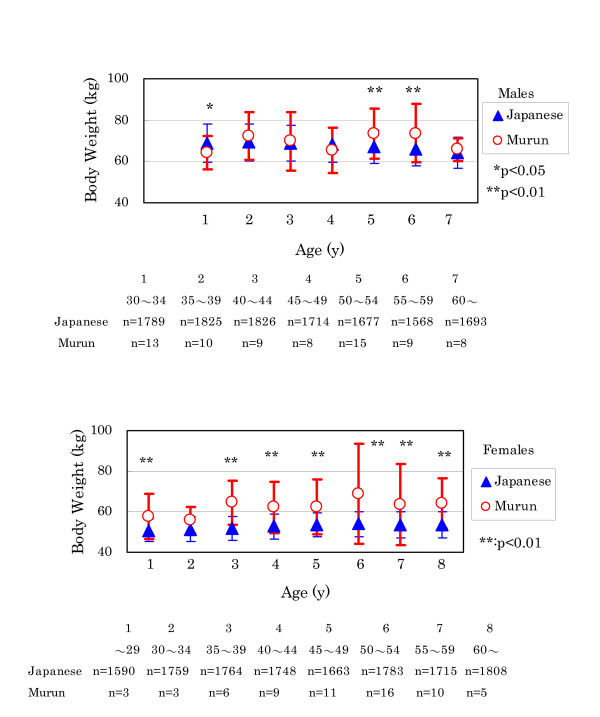
Comparison of body weight between Murun subjects and Japanese people Generally, Murun subjects showed higher body weight than age-matched Japanese people.

**Figure 4:**
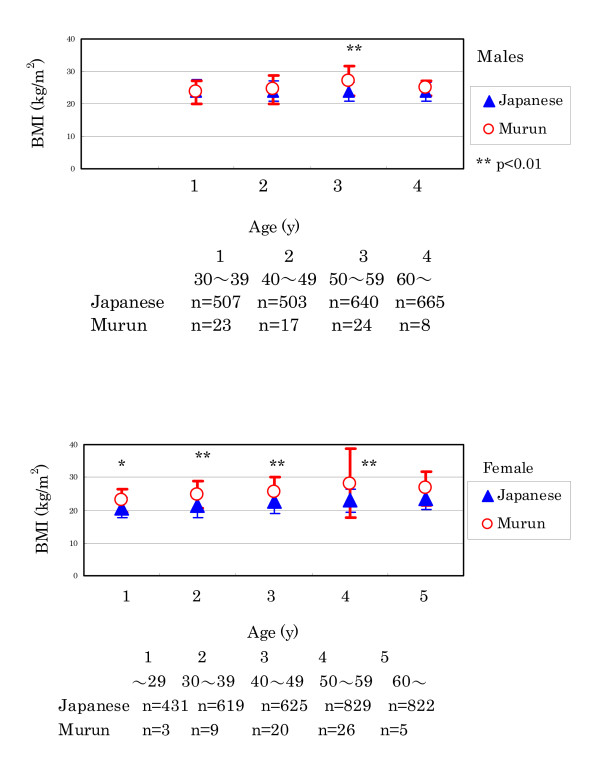
Comparison of BMI between Murun subjects and Japanese people. Murun subjects showed higher BMI levels than age-matched Japanese people. In males, the difference became clear in those over aged 50.

**Figure 5:**
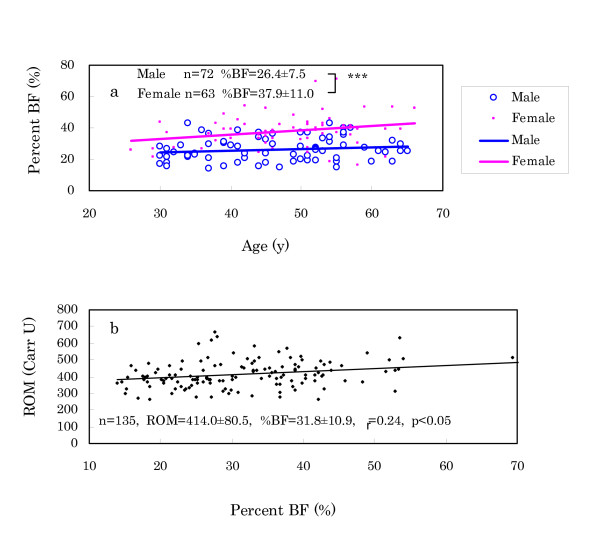
Comparison of %BF between males and females (a) and correlation of ROM levels with %BF (b). a: Females showed higher levels of %BF than males. b: This finding suggests that ROM production may have a relation with lipid accumulation.

**Figure 6:**
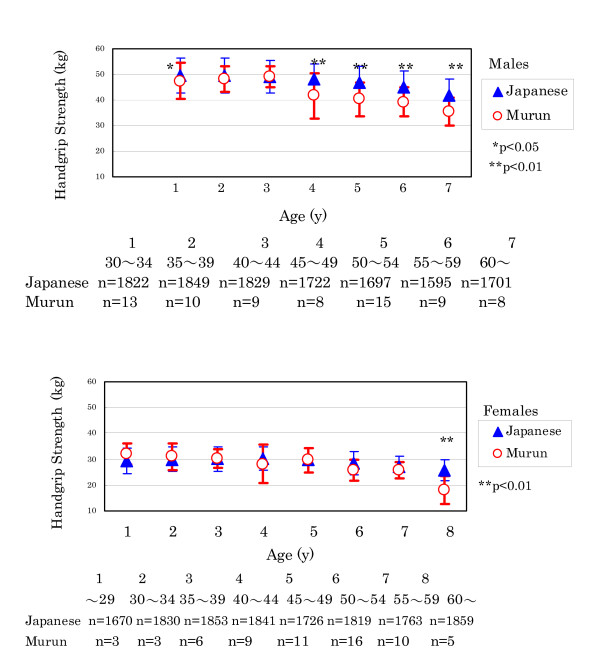
Comparison of handgrip strength between Murun subjects and Japanese people people. Handgrip strength of Murun subjects decreased earlier than that of age-matched Japanese. This finding suggests that ageing of Murun subjects may progress faster than that of Japanese people.

**Figure 7:**
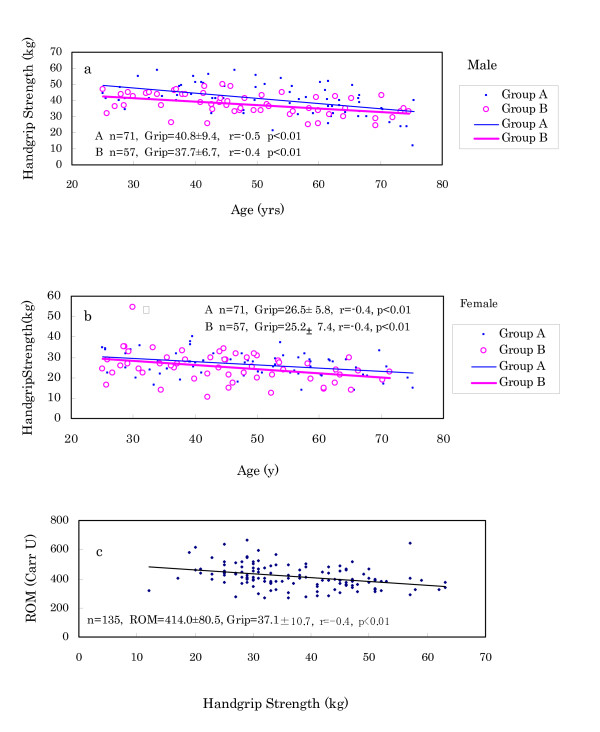
Comparison of handgrip strength between Ulaanbaatar Group A and Group B people (a and b), and correlation of ROM levels with handgrip strength in Murun subjects (c). These findings suggest that ROM production may influence the ageing process.

(1) BP and height showed no significant differences between Murun subjects and Japanese references (data not shown). But Murun subjects had higher BW than Japanese. In males, this difference was prominent for those over 50 years old (Fig. 3).

(2) Murun subjects demonstrated higher BMI levels than Japanese. In males, the difference was distinctive in 50 to 59 year-olds (Fig. 4).

(3) In regard to %BF, females showed higher levels than males (Fig. 5a). Percent BF exhibited a significant correlation with ROM levels as shown in Fig. 5b, suggesting that ROM production may have a relation to lipid accumulation.

(4) Handgrip strength of Murun subjects decreased earlier than that of Japanese references as shown in Fig. 6. In particular, Murun males over 45 years old demonstrated significantly lower levels than Japanese males, indicating that aging may proceed faster for them than for Japanese. In 2002, we measured the handgrip strength in Ulaanbaatar Group A and B people. As shown in Fig.7a and 7b, Group B people showed lower strength than Group A people. Furthermore, handgrip strength showed an inverse correlation with ROM levels, as shown in Fig. 7c, suggesting that ROM production may influence aging.

### 5. Determination of serum biochemical markers

We determined the levels of several biochemical markers using serum samples, although we could not perform the determinations in all subjects owing to the small sample volume.

(1) As described above, because BW, BMI and handgrip strength in Murun subjects largely changed in 40 to 50 year-olds, they were divided into three groups: less than 40 years, 40 to 49 years and over 50 years, and the levels of T-chol, LDL, HDL and TG were compared among these groups. As shown in Table 5, T-chol, LDL and HDL showed no significant differences among them, but the group aged 40 to 49 years showed significantly higher levels of TG than the group less than 40 years. The elevation of TG may be related to the increasing BW and BMI and decreasing handgrip strength.

**Table 5:** Serum Lipid Levels of Murum Subjects

	Age	n	T-chol	LDL	HDL	TG	ROM
	y		mg/dL	mg/dL	mg/dL	mg/dL	Carr U
Murun Males							
≦39	34.2 ± 3.1	23	182.7 ± 29.1	114.4 ± 23.9	53.6 ± 7.7	125.4 ± 99.0	369.6 ± 75.6
40~49	44.2 ± 2.7	17	178.9 ± 35.8	112.1 ± 32.9	51.7 ± 9.6	178.9 ± 35.8	366.4 ± 48.1
50≦	55.8 ± 4.7	32	182.8 ± 39.4	111.1 ± 27.5	51.3 ± 13.2	141.1 ± 117.5	390.9 ± 65.7
							
Murun Females							
≦39	33.7 ± 4.6	12	168.5 ± 22.9	103.8 ± 21.3	57.4 ± 9.6	63.7 ± 19.8	486.3 ± 82.0
40~49	44.4 ± 2.6	20	181.6 ± 36.2	104.1 ± 28.5	58.1 ± 10.1	127.3 ± 65.8	455.9 ± 75.5
50≦	55.5 ± 4.3	31	186.3 ± 27.4	111.8 ± 21.2	59.9 ± 11.2	101.1 ± 52.3	441.7 ± 74.7
							
Japanese References		1500	150–219	70–139	M 40–86	50–149	
					F 40–96		

Japanese References: the Data of a commercial Laboratory, the SRL Inc.. * p < 0.05, ** p < 0.01.

(2) The levels of serum albumin, ALT, BUN, iron, uric acid and folic acid in Murun subjects were in the same ranges as Japanese references (Table 6). Murun subjects seldom ate vegetables or fruit; thus we also measured levels of vitamins A, C and E in their blood. Vitamins A and E did not differ from the Japanese references. However, vitamin C levels were low (Table 6). The Japanese references of Table 6 are the data of a commercial laboratory (the SRL Inc., Tokyo Japan). In this study, in particular, we determined the level of vitamin C in Japanese subjects, resulting in follows; total (n = 44):11.4 ± 5.6, males (n = 26):8.7 ± 6.2, females (n = 18):14.1 ± 5.3 *μ*g/mL. This result also showed that the vitamin C levels of Murun subjects were lower than those of Japanese subjects (p < 0.001). It seems likely that these low vitamin C levels may be due to their dietary habits.

**Table 6:** Results of Biochemical Measurements in Murun Subjects.

		Japanese	Total		Males		Females	
		References	n	M ± SD	n	M ± SD	n	M ± SD
Serum								
Albumin	g/dL	3.8–5.3	135	4.3 ± 0.4	72	4.3 ± 0.4	63	4.3 ± 0.3
ALT	IU/L	5–35	135	27.2 ± 24.4	72	30.4 ± 28.1	63	24.4 ± 19.4
BUN	mg/dL	8.0–23	135	14.3 ± 4.3	72	15.1 ± 4.3	63	13.5 ± 4.1
Fe	μg/dL	M50-200, F40-180	71	104.6 ± 39.4	39	119.8 ± 39.7	32	86.0 ± 30.3
Uric acid	mg/dL	M3.8-7.5, F2.4-5.8	135	4.8 ± 1.4	72	5.4 ± 1.4	63	4.1 ± 1.0
Folic acid	ng/mL	>3.1	71	4.1 ± 1.6	39	3.7 ± 1.3	32	4.6 ± 1.8
Vitamin A	IU/dL	97–316	71	158.7 ± 56.1	39	180.6 ± 61.2	32	132.0 ± 34.3
Vitamin C	*μ*g/mL	5.5–16.8	66	3.5 ± 2.3	39	3.3 ± 2.6	27	3.7 ± 2.4
		(Japanese Subjects	44	11.4 ± 5.6	26	8.7 ± 6.2	18	14.1 ± 5.3)
Vitamin E	mg/dL	0.75–1.41	38	0.9 ± 0.3	20	0.8 ± 0.2	18	1.0 ± 0.3
MDA	U/L	Japanese Subjects	66	109.5 ± 47.7	39	117.2 ± 49.3	27	98.4 ± 43.7
		Total (n = 44) 62.5 ± 21.3						
		Males (n = 26) 74.6 ± 25.5						
		Females (n = 18) 51.8 ± 17.2						
Urine								
8-OHdG	ng/mgCre	Japanese Subjects	78	11.8 ± 3.4	47	11.3 ± 3.0	31	12.1 ± 5.7
		Total (n = 32) 6.8 ± 4.4						
		Males (n = 20) 7.5 ± 4.8						
		Females (n = 12) 5.5 ± 4.3						

ALT: alanine aminotransferase, MDA: malondialdehyde-modified LDL, BUN: blood urea nitrogen, Fe: iron, 8-OHdG: urinary 8-hydroxy-2'-deoxyguanosine Japanese References: the data of a commercial laboratory, the SRL Inc. (Tokyo, Japan). *** p < 0.001

**Figure 8:**
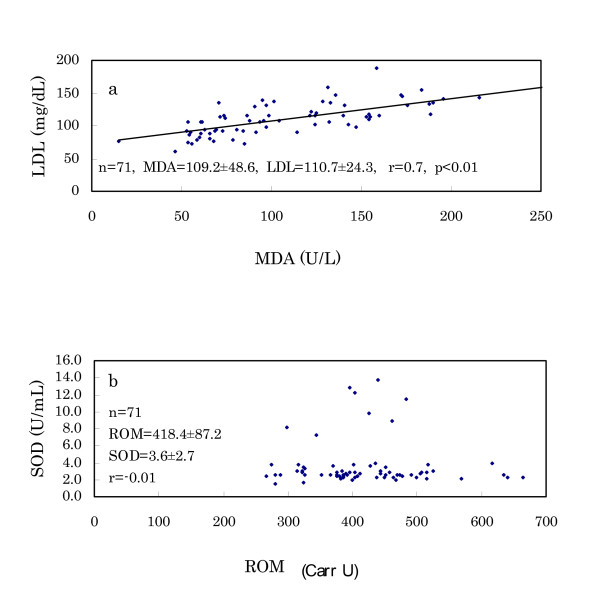
Relation of MDA levels with LDL levels (a) and SOD activities with ROM levels (b) in Murun subjects. MDA levels correlated with LDL levels significantly. SOD activities did not correlate with ROM levels, and a few subjects showed high SOD activities.

(3) The results of serum MDA and urinary 8-OHdG are also shown in Table 6. Both MDA and 8-OHdG of Murun subjects were higher than those of Japanese subjects. Although these levels of Murun subjects did not correlate with ROM levels, MDA levels were significantly correlated with LDL levels (Fig. 8a).

(4) The results of serum SOD activity are shown in Fig. 8b. Many subjects demonstrated the same levels as Japanese references (1.8–3.2 U/mL), but 8 of 72 subjects showed high activity, such as 7.8–14.1 U/mL, although the high SOD activities had no relationship to the other biochemical markers.

## Discussion

In Mongolia, non-communicable diseases, such as atherosclerosis, myocardial infarction, stroke and cancer have been increasing [17]. The short life span is a big problem in this country. In this study, we investigated oxidative stress and dietary habits in Murun subjects. Their living environment and economic conditions were not always satisfactory compared to the Ulaanbaatar Group A subjects. They seemed not to be nutritionally deficient, but they showed very high ROM levels.

Their ROM levels demonstrated the following characteristics: (a) ROM levels were very high even in young ages. (b) Because users and non-users of contraceptive pills exhibited higher ROM levels than the Japanese, other factors must also increase the ROM levels. (c) Generally, habitual heavy smokers show high oxidative stress. Smoking and its irritants enhance the infiltration of neutropohils and macrophages in lung tissue [18], and induce increased intrapulmonary oxidant burden, such as chronic obstructive pulmonary disease [19,20]. Chronic alcohol drinkers also show high oxidative stress. Trotti et al. [9] stated that ROM levels were significantly higher in heavy drinkers than in controls. But Murun subjects did not show a significant difference between smokers and non-smokers or between drinkers and non-drinkers. Therefore, other factors aside from smoking and drinking might be causing the elevation of ROM levels in Murun subjects. (d) HBV-positive and HCV-positive subjects were found at high frequencies. These hepatitis virus-positive subjects were also found in Ulaanbaatar [21]. However, no relationships between these viral infections and ROM levels were found. (e) Serum MDA and urinary 8-OHdG are important oxidative markers. MDA is produced during the degradation of lipid peroxide (22), and 8-OHdG is generated from injured DNA and excreted into urine [23,24]. Murun subjects showed higher levels of serum MDA and urinary 8-OHdG than Japanese references. These findings suggested that Murun subjects may be in high oxidative stress.

Anthropometric measurements revealed the following: (f) Murun subjects showed heavier BW and higher BMI than age-matched Japanese people. In particular, the differences became conspicuous in subjects over 50 years. These differences may be connected to increasing TG, because TG levels were high in 40 to 50 year-old subjects. (g) Percent BF was significantly correlated with ROM levels, suggesting that it may be related to ROM production. Furukawa et al. [25] found that the production of ROS increased selectively in adipose tissue of obese mice, accompanied by decreased antioxidant enzymes, and stated that oxidative stress might have an intimate relationship with fat accumulation. Keaney et al. [26] also reported that smoking, diabetes and BMI were highly associated with oxidative stress and that the oxidative stress of obesity might play an important role in cardiovascular disease. In this study, Murun females showed higher ROM levels than males. This finding may also be explained by the higher %BF in females than in males. We plan to determine the relationship among serum adiponectin, ROM levels and %BF in the next investigation. (h) Handgrip strength in the subjects over 45 years decreased more rapidly than that of age-matched Japanese. It suggested that their aging may proceed more rapidly than that of Japanese. Furthermore, because the handgrip strength was inversely correlated with ROM levels, it is likely that increasing ROM levels may accelerate aging during these years [27].

The human body naturally has AOC, which can suppress the overproduction of ROS and protect the cells from oxidative stress. AOC is composed of endogenous and exogenous elements. SOD is one of them. High oxidative stress is usually accompanied with decreasing AOC [28]. This tendency is obvious in cancer patients. In advanced cancer patients, ROM level increases and SOD activity often decreases (29). SOD may be consumed or inactivated in the high oxidative condition. However, this tendency is not always observed in other conditions. ROS is sometimes produced much more than the antioxidant systems can scavenge [12,22,30]. Furthermore, ROS often stimulate the production of antioxidant molecules. The production of SOD also increases corresponding to the high oxidative stress. Griendling et al. [31] described that ROS regulate several general classes of genes, including adhesion molecules, chemotactic factors and antioxidant enzymes, and provide an adaptive response, such as the induction of SOD and catalase. Lu et al. [32] also stated that vascular endothelial cells promptly respond to oxidative stress by synthesizing several oxidative stress-inducible proteins and stimulating antioxidant enzymes apparently to protect themselves from the toxic effects of stress. In this study, although Murun subjects demonstrated high oxidative stress, SOD activity did not always decrease. Rather, a few subjects showed high activity of SOD. Thus, the relationship of oxidative stress and SOD activity should be elucidated by re-examination in other subjects.

The dietary habits of Murun subjects were noteworthy. They mainly ate meat, wheat flour and dairy products, but seldom ate vegetables or fruit. The amounts of vegetables and fruits were fewer than those in the Ulaanbaatar Group B people. Rather, they seemed to have no habit of eating vegetables or fruit. Most Mongolian people drink koumiss in the summer. It is beneficial for obtaining vitamins. But Murun subjects did not drink it as much. It is not sufficiently produced in this area. We measured serum vitamins A, E and C levels in them. Vitamin A and E levels were the same as Japanese references, but vitamin C levels were lower. Exogenous elements of AOC are supported mainly by antioxidant foods including vitamins A, C and E, flavonoids and catechins [33]. In order to maintain AOC, it is advantageous to eat sufficient amounts of vegetables and fruits [34-36]. One of the reasons that the Murun subjects showed high ROM levels may be relevant to their dietary habits. High BMI and high %BF may also relate to their dietary habits.

Finally, as described above, it is likely that the elevation of ROM levels of Murun subjects is due to contraceptive pill use and dietary habits. Habitual smoking and alcohol drinking may also cause elevation, although the differences were not observed in this study. In addition, hard physical labor may contribute to the elevation. Strenuous exercise often induces ROS overproduction [30,37]. Chevion et al. [30] stated that very intense physical exercise leads to an increase in metabolic rate and production of ROS. Murun subjects of this study included many workers in road construction, house building, truck driving, waste cleaning, trade and cattle breeding, whereas the Japanese subjects were mainly desk workers. The hard physical labor of the Murun subjects may increase their oxidative stress. Now, the reasons why high ROM levels were observed even in young ages should be considered. There may exist other factors that induce ROM overproduction even in young subjects. In order to elucidate the factors, we are planning to measure ROM levels of the younger generation in the next study. In Mongolia, in particular, the influence of severe climate on health should also be considered. In the area of Murun city, winter temperature can go below -40°C. Olziikhutag et al. [38] performed electrocardiogram (ECG) examinations on 1357 aboriginal males of four different climatic and geographic regions of Mongolia, and found frequent nonspecific changes in T wave and ST segments. Thus, they thought that these myocardial changes might be due to the cold, as well as high altitude. Manaseki [39] and Swinburn [17] also stated that heart diseases were common in Mongolia. During 1999–2001, Mongolia experienced consecutive cold disasters that resulted in a loss of nearly six million of 33 million livestock. This disaster threatened the health and food security of approximately 40% of the country's population. As a result, growth stunting and anemia appeared in children [40]. In 2002, when we investigated the health status in Ulaanbaatar, we also found many abnormal ECG findings, including left ventricular hypertrophy. The abnormalities occurred with greater frequency in Group B than in Group A. As for the reasons, we speculated many things such as living environment, daily diets, smoking, high salt intake, hypertension, endocarditis and rheumatic heart disease due to *β*-hemolytic streptococcal infection. In particular, because Group B people had lived in nomadic areas before they moved to Ulaanbaatar, their hearts might have already been affected by the severe climate [2]. Therefore, in this study, we speculate that the severe climate may also cause physical burden and oxidative stress. In order to confirm this speculation, further examinations in different areas should be performed.

In conclusion,: Murun subjects are exposed to high oxidative stress, which may cause early aging and several non-communicable diseases, and ultimately result in the short life span of people in this country. In order to increase AOC and to suppress overproduction of ROS, contraceptive pill use, smoking and drinking should be limited. Intake of foods rich in antioxidants is also recommended. Protection against the strenuous physical activity and severe cold may also be beneficial.
